# Detection of circulating tumour cells in patients with breast or ovarian cancer by molecular cytogenetics

**DOI:** 10.1038/sj.bjc.6690825

**Published:** 1999-12

**Authors:** H Engel, C Kleespies, J Friedrich, M Breidenbach, A Kallenborn, T Schöndorf, H Kolhagen, P Mallmann

**Affiliations:** Department of Gynaecology and Obstetrics, University of Cologne, Germany; Department of Haematology and Oncology, Medical School Hannover, Carl-Neuberg-Strasse 1, 30623 Hannover, Germany

**Keywords:** circulating tumour cells, MACS, FISH

## Abstract

Detection of micrometastases in patients with solid tumours may aid the establishment of prognosis and development of new therapeutic approaches. This study was designed to investigate the presence and frequency of tumour cells in the peripheral blood (PB) of patients with breast or ovarian cancer by using a combination of magnetic activated cell sorting (MACS) and fluorescence in situ hybridization (FISH). Separated tumour cell and PB-samples from 48 patients (35 breast cancers, 12 ovarian tumours, one uterine sarcoma) were analysed for the presence of numerical aberrations of chromosomes 7, 12, 17 and 17 q11.2–q12. Twenty-five patients had primary disease and 23 had relapsed. The technique allows the detection of one tumour cell in 10^6^ normal cells. Circulating tumour cells were detected in 35/48 cases (17 patients had relapsed and 13 primary carcinoma with lymph node or solid metastases) by the expression of anti-cytokeratin and the presence of numerical chromosomal abnormalities. PB-tumour cells of patients with a primary carcinoma and without solid metastases had a significantly lower percentage of chromosomal aberrations, especially for chromosome 12 (*P* = 0.035; *P* = 0.038) compared to those with relapsed disease and solid metastases. Detection and quantification of minimal residual disease may monitor the response to cytotoxic or hormonal therapy and may identify women at risk of relapse. © 1999 Cancer Research Campaign


*British Journal of Cancer* (1999) **81**(7), 1165–1173
© 1999 Cancer Research Campaign
Article no. bjoc.1999.0825

### Detection of circulating tumour cells in patients with breast or ovarian cancer by molecular cytogenetics

**H Engel[1,2], C Kleespies[1], J Friedrich[1], M Breidenbach[1], A Kallenborn[1], T Schöndorf[1], H Kolhagen[1] and P Mallmann[1]**

[1]Department of Gynaecology and Obstetrics, University of Cologne, Germany; [2]Department of Haematology and Oncology, Medical School Hannover, Carl-Neuberg-Strasse 1, 30623 Hannover, Germany

**Summary** Detection of micrometastases in patients with solid tumours may aid the establishment of prognosis and development of new therapeutic approaches. This study was designed to investigate the presence and frequency of tumour cells in the peripheral blood (PB) of patients with breast or ovarian cancer by using a combination of magnetic activated cell sorting (MACS) and fluorescence in situ hybridization (FISH). Separated tumour cell and PB-samples from 48 patients (35 breast cancers, 12 ovarian tumours, one uterine sarcoma) were analysed for the presence of numerical aberrations of chromosomes 7, 12, 17 and 17 q11.2–q12. Twenty-five patients had primary disease and 23 had relapsed. The technique allows the detection of one tumour cell in $10^6$ normal cells. Circulating tumour cells were detected in 35/48 cases (17 patients had relapsed and 13 primary carcinoma with lymph node or solid metastases) by the expression of anti-cytokeratin and the presence of numerical chromosomal abnormalities. PB-tumour cells of patients with a primary carcinoma and without solid metastases had a significantly lower percentage of chromosomal aberrations, especially for chromosome 12 ($P = 0.035$; $P = 0.038$) compared to those with relapsed disease and solid metastases. Detection and quantification of minimal residual disease may monitor the response to cytotoxic or hormonal therapy and may identify women at risk of relapse. © 1999 Cancer Research Campaign

**Keywords:** circulating tumour cells; MACS; FISH

The detection and elimination of minimal disseminated disease in patients with solid tumours is one of the main current topics in clinical oncology (Pantel, 1996).

A variety of assays of widely varying sensitivity have been utilized for the detection of circulating tumour cells such as light microscopy, cytogenetic analyses, fluorescence in situ hybridization (FISH), Southern blot analysis, immunocytochemistry, polymerase chain reaction (PCR) and flow cytometry (Kvalheim, 1996; Pantel, 1996; Vrendenburgh et al, 1996; Schoenfeld et al, 1997; Traystman et al, 1997).

Because of the fact that breast and ovarian cancers do not appear to have tumour-specific chromosomal aberrations, tumour cell detection by molecular methods is based on the amplification of tissue-specific transcripts (Mapara et al, 1997; Bostick et al, 1998). In immunocytochemical assays, epithelial specific antibodies have been used to detect isolated tumour cells in bone marrow (BM) and blood (Cote et al, 1991, 1995, 1996; Diel et al, 1996; Franklin et al, 1996).

In an effort to obtain greater sensitivity, several investigators have developed techniques for the enrichment of tumour cells before their identification by immunocytochemistry, PCR or FISH (Hardingham et al, 1993, 1995; Berois et al, 1997; Eaton et al, 1997; Hildebrandt et al, 1997; Naume et al, 1997; Martin et al, 1998).

Therefore, the aim of our study was to analyse the presence and frequency of circulating tumour cells in the peripheral blood of patients with breast or ovarian cancer by using a combination of magnetic activated cell sorting (MACS) and interphase FISH.

*Received 7 October 1998*
*Revised 17 May 1999*
*Accepted 25 May 1999*

*Correspondence to:* H Engel

### MATERIALS AND METHODS

### Patients

In this study we included 48 adult patients with a median age of 60.6 ± 13.3 years (range 30–86). Thirty-five had the diagnosis of breast cancer, 12 ovarian cancer and one uterine sarcoma. Twenty-five patients had primary disease (6/25 without involvement of axillary lymph nodes, 8/25 with solid metastases, 11/25 with lymph node metastases) and 23 had relapsed (20/23 with solid metastases). PB-samples were obtained following informed consent at the time of diagnosis, during or after therapy. MACS-sorted tumour cells were analysed by interphase FISH, using α-satellite probes specific for the centromeric regions of chromosomes 7, 12, 17 and the region 17 q11.2–q12 (HER-2/neu). Controls were PB-samples from five normal volunteers to determine the background for each probe.

### Magnetic cell separation

Mononuclear cells were isolated from fresh blood by Ficoll-Hypaque gradient separation. After washing in PBS per 5 mm EDTA, 300 μl PBS buffer per $10^8$ cells, 20 μl of CK-8 microbeads (Miltenyi Biotec GmbH, Bergisch Gladbach, Germany) were added. After gentle mixing and incubation for 15 min at 4°C, the cells were washed once in 5 ml buffer per $10^8$ cells. The buffer was completely removed, and the cells were resuspended again in 400 μl buffer. The cell suspension was then applied to a prefilled

**Table 1** Anatomical site, pathology and percentage of chromosomal aberrations in PB-tumour cells from 45 patients with gynaecological cancer

| Patient | Diagnosis | Stage | Grade | Metastases | Aberrant cells (%) | | |
|---|---|---|---|---|---|---|---|
| no. | | | | | Chromosome 7 | Chromosome 12 | Chromosome 17 |
| 1 | Breast cancer (relapse) | I | G I | Solid | 7.0 | 4.7 | 17.5 |
| 2 | Primary breast cancer | I | ND | None | | | |
| 2a | | | | | 3.0 (negative[a]) | 3.7 (negative[a]) | 14.3 |
| 2b | | | | | 6.3 | 3.7 (negative[a]) | 15.3 |
| 2c | | | | | 7.6 | 11.0 | 13.3 |
| 2d | | | | | 6.0 | 3.0 (negative[a]) | 10.5 |
| 3 | Breast cancer (relapse) | I | G II | Lymph nodes | 5.3 (negative[a]) | 9.0 | 14.0 |
| 4 | Ovarian cancer (relapse) | II | ND | Solid | 5.7 | 5.7 (negative[a]) | 12.0 |
| 5 | Breast cancer (relapse) | I | G II | Solid | 4.3 (negative[a]) | 6.5 (negative[a]) | ND |
| 6 | Ovarian cancer (relapse) | I | ND | Solid | 2.7 (negative[a]) | 5.7 (negative[a]) | 15.5 |
| 7 | Primary ovarian cancer | IV | ND | Solid | 5.1 (negative[a]) | 4.7 (negative[a]) | 11.4 |
| 8 | Primary breast cancer | II | ND | Lymph nodes | 4.0 (negative[a]) | 5.3 (negative[a]) | 8.7 (negative[a]) |
| 9 | Ovarian cancer (relapse) | III | ND | Solid | 4.7 (negative[a]) | 7.3 | 12.3 |
| 10 | Primary breast cancer | II | G III | Lymph nodes | 6.3 | 6.3 (negative[a]) | 14.3 |
| 11 | Breast cancer (relapse) | IV | ND | Solid | 4.0 (negative[a]) | 11.0 | 10.7 |
| 12 | Primary breast cancer | I | G II | Lymph nodes | ND | ND | 18.0 |
| 13 | Breast cancer (relapse) | III | ND | Solid | 4.7 (negative[a]) | 5.7 (negative[a]) | 8.6 (negative[a]) |
| 14 | Primary breast cancer | II | G II | Lymph nodes | 7.7 | 12.0 | 16.0 |
| 15 | Primary breast cancer | IV | G II | Solid | 5.0 (negative[a]) | 5.7 (negative[a]) | 11.0 |
| 16 | Breast cancer (relapse) | II | G III | Solid | 3.7 (negative[a]) | 8.3 | 17.0 |
| 17 | Primary breast cancer | I | G II | Lymph nodes | 0 | 8.5 | ND |
| 18 | Ovarian cancer (relapse) | IV | G II | Solid | 2.3 (negative[a]) | 13.0 | 22.0 |
| 19 | Primary breast cancer | IV | ND | Lymph nodes | ND | ND[1] | 4.2 (negative[a]) |
| 20 | Breast cancer (relapse) | I | ND | Solid | 28.1 | 22.8 | ND |
| 21 | Primary breast cancer | II | G I | None | ND | 4.4 (negative[a]) | ND |
| 22 | Ovarian cancer (relapse) | IV | ND | Solid | 0 | 0 | ND |
| 23 | Breast cancer (relapse) | IV | G I | None | 4.0 (negative[a]) | 10.0 | ND |
| 24 | Ovarian cancer (relapse) | III | ND | Solid | 1.3 (negative[a]) | 6.7 | 9.7 |
| 25 | Breast cancer (relapse) | IV | ND | Solid | 4.8 (negative[a]) | 6.0 (negative[a]) | ND |
| 26 | Primary breast cancer | IV | ND | Solid | 13.0 | 15.0 | ND |
| 27 | Primary breast cancer | II | ND | Solid | 5.0 (negative[a]) | 6.0 (negative[a]) | 14.5 |
| 28 | Breast cancer (relapse) | II | G II | Solid | 7.6 | 15.1 | ND |
| 29 | Primary breast cancer | II | G II | None | 7.3 | 5.3 (negative[a]) | 14.7 |
| 30 | Primary breast cancer | II | ND | Solid | 3.0 (negative[a]) | 3.0 (negative[a]) | 13.0 |
| 31 | Primary breast cancer | I | ND | None | 4.7 (negative[a]) | 3.0 (negative[a]) | 14.0 |
| 32 | Primary breast cancer | I | G II | Lymph nodes | 7.0 | 7.0 | 10.0 |
| 33 | Primary breast cancer | IV | ND | Lymph nodes | ND | ND | 13.7 |
| 34 | Primary ovarian cancer | III | G II | Solid | 1.5 (negative[a]) | 4.6 (negative[a]) | ND |
| 35 | Primary ovarian cancer | III | ND | Solid | 6.0 | 11.3 | 14.3 |
| 36 | Breast cancer (relapse) | I | G III | None | 7.0 | 9.3 | 11.5 |
| 37 | Primary ovarian cancer | II | G II | Lymph nodes | 0 | 3.4 (negative[a]) | ND |
| 38 | Primary breast cancer | II | G III | Solid | 1.3 (negative[a]) | 9.3 | 10.5 |
| 39 | Primary breast cancer | I | ND | None | 3.3 (negative[a]) | 3.7 (negative[a]) | 10.7 |
| 40 | Primary breast cancer | I | ND | None | 5.5 | 5.5 (negative[a]) | 10.7 |
| 41 | Ovarian cancer (relapse) | III | ND | Solid | 0 | 7.0 | ND |
| 42 | Breast cancer (relapse) | II | ND | Solid | 0 | 10.0 | ND |
| 43 | Uterus sarcoma (relapse) | II | G III | Solid | 6.0 | 18.0 | ND |
| 44 | Ovarian cancer (relapse) | IV | ND | Solid | 4.9 (negative[a]) | 9.2 | ND |
| 45 | Primary breast cancer | I | G III | Lymph nodes | negative | negative | negative |
| 46 | Primary breast cancer | I | G II | Lymph nodes | negative | negative | negative |
| 47 | Breast cancer (relapse) | II | ND | Solid | negative | negative | negative |
| 48 | Breast cancer (relapse) | II | ND | Solid | negative | negative | negative |
| | | | | Mean ± s.e.m. | 5.0 ± 0.7 | 7.7 ±0.7 | 12.9 ± 0.6 |

ND, not determined; [a]Percentage of chromosomal aberrations less than background (mean + 3 SD of normal control cells)

MiniMACS column [MS$^+$RS$^+$ or MACS VS$^+$ separation columns (Miltenyi Biotec GmbH, Bergisch Gladbach, Germany)]. The cells were passed through the column and washed four times with 500 µl buffer. The column was removed from the separator, and the CK-enriched cells were eluted using the plunger.

### Flow cytometry

The following directly conjugated antibodies [anti-cytokeratin (Dako AS, Glostrup, Denmark) and anti-CD45 (Becton Dickinson, Heidelberg, Germany)] were used to detect circulating

*© 1999 Cancer Research Campaign*

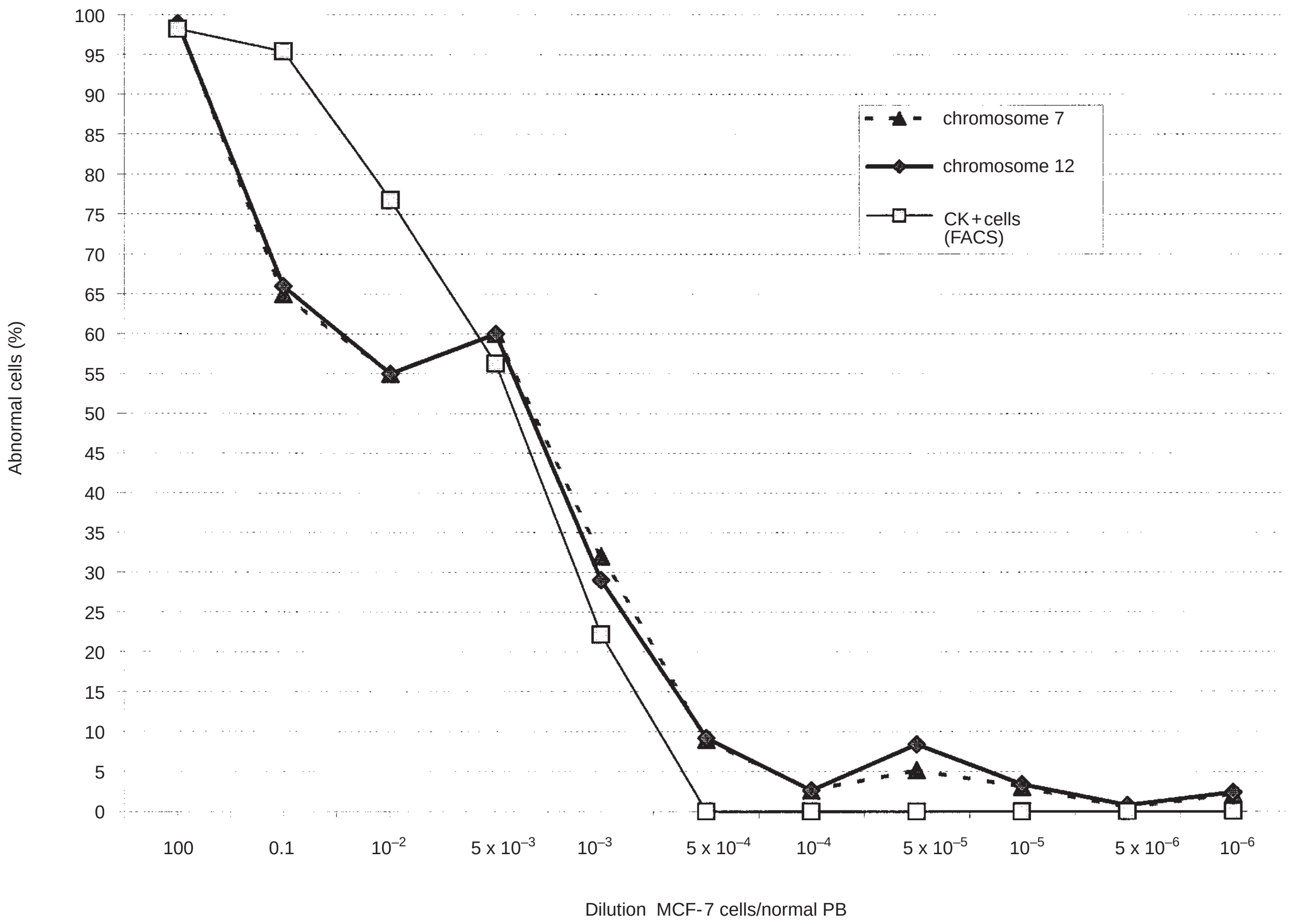

**Figure 1** Dilution experiment of normal peripheral blood containing MCF-7 cells. Flow cytometry after MACS-sorting using an anti-cytokeratin and an anti-CD45 antibody failed to detect tumour cells at the $5 \times 10^{-4}$ level. By applying FISH after MACS-sorting using chromosome probes for the centromeric region of chromosomes 7 and 12 detection of tumour cells in a dilution as few as $1 \times 10^{-6}$

tumour cells. A total of 10 µl of anti-CD45 PerCP (peridinin–chlorophyll–protein) conjugated antibody were added to $1 \times 10^6$ mononuclear cells and incubated for 15 min at room temperature in darkness. Appropriate isotype controls were used to set the amplification and compensation of the flow cytometer. The cells were then washed twice with PBS and fixed in 1 ml 0.25% paraformaldehyde at room temperature for 15 min. After another wash with PBS, cells were incubated in cold (4°C) 70% methanol for 60 min in darkness before staining with 10 µl anti-cytokeratin FITC (fluorescein isothiocyanate)-labelled antibody. After an incubation of 30 min at 4°C, the cells were washed with PBS, resuspended in 300 µl PBS and then analysed with a FACScalibur flow cytometer (Becton Dickinson, San Jose, CA, USA). Analysis by flow cytometry was done before and after MACS-sorting. Tumour cells were defined as cytokeratin$^+$/CD45$^-$.

### Fluorescence in situ hybridization

Sorted cells were fixed in 3:1 methanol:glacial acetic acid and stored until hybridization at –20°C. Slides were incubated at room temperature in 0.1 N hydrochloric acid with 0.05% Triton X-100 for 15 min and then washed six times: once for 2 min in 2 × saline sodium citrate (SSC; 0.3 M sodium chloride, 30 mM sodium citrate, pH 7), once in PBS, once in PBS with 1% formaldehyde for 5 min, twice for 2 min with PBS, and finally once with 2 × SSC. Slides were denatured in 70% formamide in 2 × SSC at 70°C for 2–4 min, dehydrated in a 70%, 85%, 100% ethanol series and air-dried.

Directly conjugated centromeric probes (CEP) specific for chromosomes 7, 12 and 17 (Vysis, Stuttgart, Germany) were used for interphase FISH (CEP 7 conjugated to Spectrum Green, CEP 12 and 17 conjugated to Spectrum Orange, CEP 17/17q.11.2–q12 conjugated to Spectrum Green/Orange). One microlitre of each probe was mixed with 7 µl hybridization buffer (50% formamide, 2 × SSC, 10% dextran sulphate) and 2 µl distilled water. Probe DNA was denatured for 5 min at 70°C and applied to each slide. Hybridization was performed overnight at 37°C in a humidified chamber.

Non-hybridized probe was washed off by a series of three post-hybridization washes in 50% formamide in 2 × SSC at 45°C each for 10 min, followed by one 10-minute wash in 2 × SSC and one 5-min wash in 2 × SSC/0.1% NP-40 at 37°C. The nuclei were counterstained with diamino-2-phenylindole dihydrochloride (DAPI, 0.2 µM in 90% glycerol/10% PBS, pH 8.0). Hybridization signals were counted by hand in 100–500 cells under a fluorescence microscope (Leica, Heerbrugg, Switzerland) equipped with a triple filter set (DAPI/FITC/Texas-Red).

© 1999 Cancer Research Campaign

**Table 2** Dilution experiment of normal peripheral blood containing MCF-7 cells

| Dilution MCF-7/nPB[a] | Chromosome 7[b] (% abnormal cells)[c] | Chromosome 12[b] (% abnormal cells)[c] | Cytokeratin+ cells[d] (Flow cytometry) |
|---|---|---|---|
| 100 | 98.7 | 98.4 | 98.2 |
| 0.1 | 65 | 66 | 95.4 |
| $10^{-2}$ | 55 | 55 | 76.8 |
| $5 \times 10^{-3}$ | 60 | 60 | 56.3 |
| $10^{-3}$ | 32 | 29 | 22.2 |
| $5 \times 10^{-4}$ | 9 | 9.2 | 0 |
| $10^{-4}$ | 2.7 | 2.7 | 0 |
| $5 \times 10^{-5}$ | 5.2 | 8.4 | 0 |
| $10^{-5}$ | 3.1 | 3.4 | 0 |
| $5 \times 10^{-6}$ | 0.5 | 0.8 | 0 |
| $10^{-6}$ | 2.2[e] | 2.4[f] | 0 |

[a]nPB; normal peripheral blood; [b]at least 300 cells were counted; [c]cells with trisomies or tetrasomies; [d]10 000 events were acquired; [e]background trisomy 7: 1.32%; [f]background trisomy 12: 1.10%.

### Statistical analysis

Data are expressed as mean (± s.e.m.) or as mean (± s.d.) and analysed by using the two-way analysis of variance and other standard methods. The SPSS statistical package was employed for these analyses as well as to generate descriptive statistics of the data.

### RESULTS

We investigated 48 patients with gynaecological cancer (Table 1). To obtain greater sensitivity, we applied FISH on MACS-sorted cells. To distinguish monosomy and trisomy from background, cut-off levels were set at 3 standard deviations (s.d.) above the mean percentages of normal control cells ($n = 5$) with one or three signals (> 5.3% for abnormalities of chromosome 7, > 6.3% for abnormalities of chromosome 12, > 8.2% for abnormalities of chromosome 17).

### FISH on primary tumours and cell lines

As a positive control for aberrations of chromosomes 7, 12 and 17 we analysed a breast cancer (MCF-7) and an ovarian cancer cell line (MZ-1b). All MCF-7 and MZ-1b cells were aberrant for chromosomes 7 and 12 and 87.5% of these cells showed numerical abnormalities for chromosome 17. SK-BR-3 human breast cancer cells (97% of aberrant tumour-cell nuclei) were used as a control for Her-2/neu amplification.

Tumour cells obtained from tissue or effusions ($n = 10$) showed a high frequency of chromosomal aberrations (an average of 45%) mainly trisomies and tetrasomies of chromosomes 7, 12 and 17. A monosomy pattern was limited to chromosome 17. Her-2/neu amplification was identified on average in 36% of tumour-cell nuclei with high copy levels.

### Sensitivity

Dilution experiments were performed to determine the sensitivity of FISH after MACS-sorting. MCF-7 cells with an average of 98% of numerical aberrations for chromosomes 7 and 12 (trisomies and tetrasomies) were sorted into normal peripheral blood (0.1–$10^{-6}$). By comparing flow cytometry after MACS-sorting using an anti-cytokeratin and an anti-CD45 antibody and FISH ($\alpha$-satellite probes for chromosomes 7, 12 and 17), flow cytometry failed to detect tumour cells at a level of $5 \times 10^{-4}$. With FISH we were able to identify 2.4% of chromosomal aberrant cells in a dilution containing as few as 1 tumour cell in $10^6$ normal cells (Figure 1 and Table 2).

### Flow cytometry after MACS-sorting of PB-tumour cells

On 12 samples from 9/25 patients with primary carcinoma, we applied flow cytometry after MACS-sorting by doublestaining with anti-FITC-conjugated anti-cytokeratin and anti-PerCP-conjugated CD45 antibody. Tumour cells were defined as $CK^+/CD45^-$. The purity ranged from 0.04% to 18.50% (median: 7.30%; Table 3 and Figure 2). Some cells even double expressed CK and CD45, suggesting a false-positive detection of tumour cells. There was no correlation between sorting purity and the frequency of chromosomal aberrations detected by FISH.

**Table 3** Flow cytometry and FISH including HER-2/neu after MACS-sorting of PB-tumour cells from 10/28 patients with primary carcinoma

| Patient no. | CK + (%) | Aberrant cells (%) | | | |
|---|---|---|---|---|---|
| | | Chromosome 7 | Chromosome 12 | Chromosome 17 | Her-2/neu |
| 2a | 18.5 | 3.0 (negative[a]) | 3.7 (negative[a]) | 14.3 | 18.8 |
| 2b | 10.8 | 6.3 | 3.7 | 15.3 | 16.7 |
| 2c | 13.3 | 7.6 | 11.0 | ND | 36.4 |
| 2d | 1.92 | 6.0 | 3.0 (negative[a]) | 10.5 | negative |
| 12 | 0.08 | ND | ND | 18.0 | 46.2 |
| 17 | 2.8 | 0 | 8.5 | ND | ND |
| 19 | 0.04 | ND | ND | 4.2 (negative[a]) | 22.7 |
| 21 | 7.3 | ND | 4.4 (negative[a]) | ND | ND |
| 30 | 10.0 | 3.0 | 3.0 (negative[a]) | 13.0 | ND |
| 33 | 7.7 | ND | ND | 13.7 | 41.5 |
| 34 | 13.0 | 1.5 | 4.6 (negative[a]) | ND | ND |
| 41 | 2.1 | 0 | 7.0 | ND | ND |
| Mean ± s.e.m. | 6.9 ± 1.6 | 3.4 ± 1.0 | 5.4 ± 0.9 | 12.7 ± 1.7 | 30.4 ± 5.1 |

ND, not determined; [a]Percentage of chromosomal aberrations less than background (mean + 3 SD of normal control cells)

 *© 1999 Cancer Research Campaign*

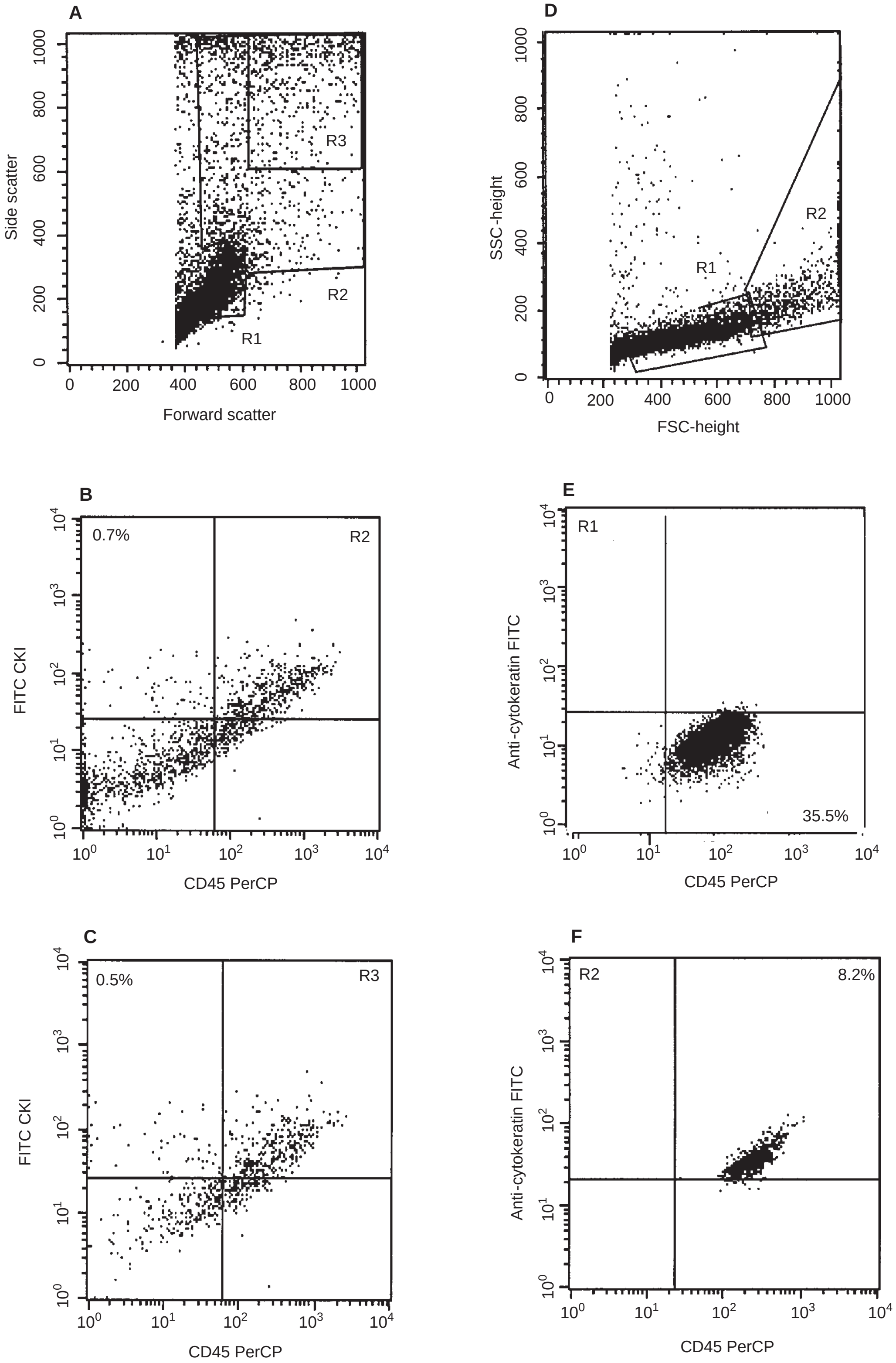

**Figure 2** Flow cytometry before and after MACS-sorting of peripheral blood cells. Application of flow cytometry before (**A**–**C**) and after MACS-sorting (**D**–**F**) by doublestaining with anti-FITC conjugated anti-cytokeratin and anti-PerCP-conjugated CD45 antibody. Tumour cells were defined as $CK^+/CD45^-$. Before MACS-sorting (**B**, **C**) 0.7% of $CK^+/CD45^-$ cells (R2 and R3). After enrichment a high contamination with leucocytes (**E**); all cells located in R2 expressed CK but were also $CD45^+$ (F), suggesting a high rate of false-positive results

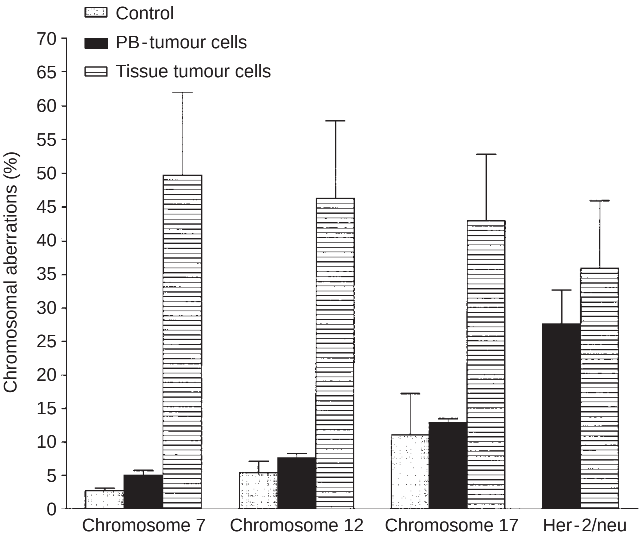

**Figure 3** Percentage of chromosomal aberrations in PB-tumour cells compared to tissue tumour cells. Tissue tumour cells showed a much higher frequency of numerical aberrations for chromosomes 7, 12, 17 and 17q compared to PB-tumour cells, which had only high Her-2/neu amplification levels

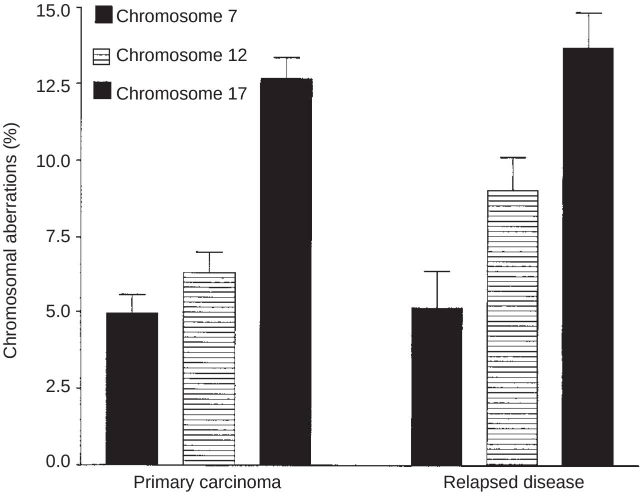

**Figure 5** Chromosomal aberrations of PB-tumour cells from patients with primary carcinoma or relapsed disease. PB-tumour cells of patients with a primary carcinoma had a significantly lower percentage of chromosomal aberrations, especially for chromosome 12 ($P$ = 0.035) compared to those with relapsed disease

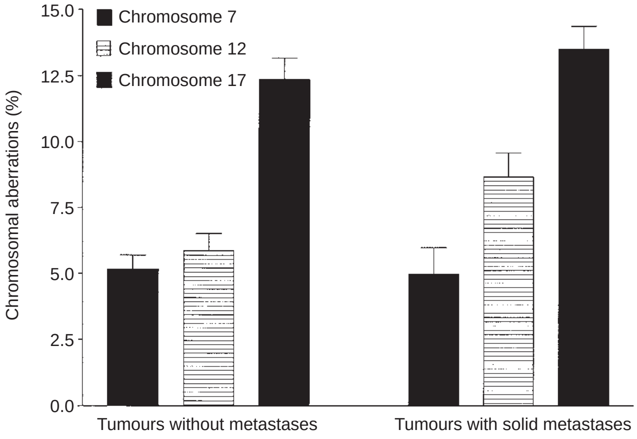

**Figure 4** Comparison of chromosomal aberrations of PB-tumour cells from patients with/without solid metastases. PB-tumour cells of patients without solid metastases had a significantly higher percentage of chromosomal aberrations for chromosome 12 ($P$ = 0.038) than patients without solid metastases

### FISH on MACS-sorted PB-tumour cells

In 35/48 peripheral blood samples (17 patients had relapsed and 13 primary carcinoma with lymph node or solid metastases) we were able to detect tumour cells which carried chromosomal aberrations; they were identified by the fluorescence pattern of their nuclei (Table 1 and Figure 3). To confirm the presence of tumour cells after MACS-sorting, cells were doublestained for anti-cytokeratin and anti-CD45. These cells showed aberrations appearing mainly as monosomies for chromosome 17 (12.93% ± 0.59%) and as trisomies for chromosomes 7 and 12 (5.04% ± 0.68% and 7.64% ± 0.66%). Tumour cells from six PB-samples (primary carcinoma) were also studied for amplification of the region 17q11.2–q12 (Table 3 and Figure 3). The frequency of aberrations (30.36% ± 5.13%) was much higher than numerical changes of chromosomes 7, 12 and 17.

PB-tumour cells from patients with a primary carcinoma without solid metastases had a significantly lower percentage of chromosomal aberrations, especially for chromosome 12 ($P$ = 0.035; $P$ = 0.038) compared to those with relapsed disease and solid metastases (Figures 4 and 5). There was no statistically significant difference in the frequency of chromosomal aberrations with respect to lymph node involvement in patients with primary carcinoma. Twelve out of 25 patients with a primary carcinoma are still in clinical remission with a median follow-up of 20 months (range 4–24 months), one patient died in clinical remission (CR) after 2 months, four relapsed after a median CR duration of 11 months (range 5–12 months). There was no correlation between the frequency of chromosomal aberration in PB-tumour cells and remission duration. Five of 8 patients with primary carcinoma and solid metastases have stable disease with a median follow-up of 19 months (range 9–22 months), one patient has progressive disease and two patients died shortly after diagnosis because of their metastases.

In 13/48 samples (seven patients with primary carcinoma and four of them without lymph node involvement, six patients with relapsed disease and solid metastases) we could not detect tumour cells either by flow cytometry or by FISH. The six patients with solid metastases are clinically unchanged (median follow-up 24 months); three patients are in CR (median follow-up months), one patient died after 2 months in CR and three patients relapsed after a median remission duration of 5 months.

### Tumour cell detection in peripheral blood and bone marrow: a follow-up of a patient with primary breast cancer

Four PB and one BM sample were obtained from a patient with primary breast cancer (patient no. 2, Tables 1 and 3) at diagnosis and at three different time points after surgery. The patient did not receive any chemotherapy or hormonal treatment.

 © 1999 Cancer Research Campaign

Cells were doublestained for anti-cytokeratin and anti-CD45 before MACS-sorting. By flow cytometry we were able to detect 23 tumour cells $\mu l^{-1}$ in the first PB-sample (obtained before tumour resection), one tumour cell $\mu l^{-1}$ 4 months after surgery, two tumour cells $\mu l^{-1}$ in a follow-up sample 8 months after diagnosis compared to 104 tumour cells $\mu l^{-1}$ in the bone marrow. We could not detect any $CK^{+}/CD45^{-}$ cells in a PB-sample obtained 14 months after diagnosis. However, by applying FISH after MACS-sorting we identified peripheral blood tumour cells with aberrations for chromosome 7 (5.8% ± 1.0%), chromosome 12 (5.3% ± 1.9%), chromosome 17 (13.4% ± 1.0%) and for the region 17q11.2–q12 (23.9% ± 6.3%) without any statistically significant differences between the first three samples (Tables 1 and 2). The frequency of aberrant tumour cells was lower in the fourth sample and no Her-2/neu amplification could be detected. BM tumour cells showed in 11.8%, numerical aberrations for the centromeric region of chromosome 17 and amplification for Her-2/neu in 18.2%.

### DISCUSSION

The detection of small numbers of carcinoma cells in patients with solid tumours has become increasingly important and may help in determining the prognosis and the development of new therapeutic approaches (Datta et al, 1994; Ross, 1998).

Many techniques such as immunocytochemistry, flow cytometry, PCR and FISH have been used to detect micrometastases in peripheral blood, bone marrow, aphereses or lymph nodes (Smith et al, 1991; Kvalheim, 1996; Pantel, 1996; Vrendenburgh et al, 1996; Schoenfeld et al, 1997; Traystman et al, 1997).

In immunocytochemical assays, monoclonal antibodies to cytokeratins can be regard as a sensitive probe to detect isolated epithelial tumour cells in bone marrow and blood (Diel et al, 1996; Franklin et al, 1996).

Molecular methods are based on the detection of either mutations in oncogenes and tumour suppressor genes or mRNA expression of tissue-specific and tumour-associated genes (Datta et al, 1994; Schoenfeld et al, 1994, 1997; Kruger et al, 1996; Luppi et al, 1996; Moscinski et al, 1996; Noguchi et al, 1996). Nevertheless, the immunocytochemistry method needs to be further developed before it can be used routinely in the clinic and it is not clear whether the most frequently employed reverse transcription PCR (RT-PCR) assays for cytokeratin 18 or 19 or pancarcinoma-associated tumour marker (KSA or 17-1A antigen) have the specificity to be reliably used (Kvalheim, 1996; Helfrich et al, 1997).

Human breast and ovarian cancers appear, despite their considerable pathologic uniformity, to be heterogeneous with respect to biological and clinical behaviour and these tumours are not associated with unique karyotypic changes (Deville et al, 1988). Conventional cytogenetic studies and FISH analyses of breast and ovarian tumour cells have shown multiple chromosomal abnormalities involving chromosomes 7, 12 and 17 (Dutrillaux et al, 1990; Geleick et al, 1990; Cajulis et al, 1994; Persons 1994; Xu et al, 1994; Fiegl et al, 1995; Visscher et al, 1995, 1996; Ishikawa et al, 1996, Engel et al, 1998). The erbB-2 oncogene located on chromosome 17q is expressed in a substantial number of breast tumours and associated with a poor prognosis (Kallioniemi et al, 1992; An et al, 1995; Schildkraut et al, 1995; Fernandez et al, 1996; Sauter et al, 1996; Ishikawa et al, 1997). Recent studies (Press et al, 1997; Revillion et al, 1998) have confirmed a significantly worse survival of erbB-2-positive patients and suggest that erbB-2 could be a marker of reduced response to chemotherapy and hormonal treatment.

Fluorescence in situ hybridization, by which many cells can be screened, independent of their capacity to proliferate in vitro, has become a complementary tool in cancer cytogenetics for the detection of numerical aberrations in interphase nuclei and for the classification of marker chromosomes (Kiechle-Schwarz et al, 1991; Le Beau, 1993; Micale et al, 1994; Muller et al, 1996).

The immunomagnetic MACS system, using magnetic beads coated with a cocktail of monoclonal antibodies recognizing the leucocyte common antigen CD45 or the CK-antigen developed by Miltenyi et al in 1990, is an extremely efficient method for separating cells (Harbeck et al, 1995). No morphological alterations were observed after the separation, which suggests that the passage through a strong magnetic field does not damage the cells.

In this study, we used FISH on MACS-sorted tumour cells to investigate the presence and frequency of micrometastases in patients with breast or ovarian cancer by using CK-8 microbeads and α-satellite probes specific for the centromeric regions of chromosomes 7, 12 and 17 and the region 17q11.2–q12 (HER-2/neu).

Trisomies and tetrasomies of chromosomes 7, 12 and 17, as well as combined aberrations, have been identified by FISH in a substantial number of tumour cells obtained from tumour tissue. A monosomy pattern was limited primarily to chromosome 17, thus correlating with previous cytogenetic studies (Visscher et al, 1996; Engel et al, 1998).

Circulating tumour cells were present in 35/48 peripheral blood samples. Flow cytometry after MACS-sorting failed at a detection level of $5 \times 10^{-4}$. The sorting purity ranged from 0.04% to 18.50% in patients samples, suggesting a low degree of specificity and substantial contamination with normal PB lymphocytes. FISH after MACS-sorting is an alternative method for detection of circulating tumour cells with a higher sensitivity (one tumour cells in $10^{6}$ normal cells). However, PB-tumour cells carried aberrations for chromosomes 7, 12 and 17 as well but at a much lower frequency (especially for chromosomes 7 and 12) compared to those obtained from tumour tissue. In fact, these very low rates of aberrations might suggest that chromosomes 7 and 12 are less frequently involved in micrometastases of patients with breast or ovarian cancer. On the other hand, it is tempting to speculate that PB-tumour cells might be different with respect to their biological behaviour. Nevertheless, higher levels of Her-2/neu copies (on average 30%) and numerical aberrations for the centromeric region of chromosome 17 (on average 13%) were detected, suggesting chromosome 17 is frequently involved and might be a sensitive marker for the detection of circulating tumour cells. However, follow-up samples at different time points in clinical remission are necessary to prove the value of circulating tumour cells.

The technique described could become a valuable tool for the quantification and molecular characterization of metastatic carcinoma cells and might provide the basis to monitor the response to cytotoxic or hormonal therapy and may identify women at risk of relapse.

### REFERENCES

An HX, Niederacher D, Beckmann MW, Gohring UJ, Scharl A, Picard F, van Roeyen C, Schnurch HG and Bender HG (1995) ERBB2 gene amplification detected by fluorescent differential polymerase chain reaction in paraffin-embedded breast carcinoma tissues. *Int J Cancer* **64**: 291–297

*© 1999 Cancer Research Campaign* 

Berois N, Varangot M, Osinaga E, Babino A, Caignault L, Muse I and Roseto A (1997) Detection of rare human breast cancer cells. Comparison of an immunomagnetic separation method with immunocytochemistry and RT-PCR. *Anticancer Res* **17**: 2639–2646

Bostick PJ, Chatterjee S, Chi DD, Huynh KT, Giuliano AE, Cote R and Hoon DS (1998) Limitations of specific reverse-transcriptase polymerase chain reaction markers in the detection of metastases in the lymph nodes and blood of breast cancer patients. *J Clin Oncol* **16**: 2632–2640

Cajulis RS, Kotliar S, Haines GK, Frias-Hidvegi D and O'Gorman M (1994) Comparative study of interphase cytogenetics, flow cytometric analysis, and nuclear grade of fine-needle aspirates of breast carcinoma. *Diagn Cytopathol* **11**: 151–158

Datta YH, Adams PT, Drobyski WR, Ethier SP, Terry VH and Roth MS (1994) Sensitive detection of occult breast cancer by the reverse-transcriptase polymerase chain reaction. *J Clin Oncol* **12**: 475–482

Deville P, Theirry RF, Kievitis T, Kolluri R, Hopman AHN, Willard HF, Pearson PL and Cornelisse CJ (1988) Detection of chromosome aneuploidy in interphase nuclei from human primary breast tumors using chromosome-specific repetitive DNA probes. *Cancer Res* **48**: 5825–5830

Cote RJ, Rosen PP, Lesser ML, Old LJ and Osborne MP (1991) Prediction of early relapse in patients with operable breast cancer by detection of occult bone marrow micrometastases. *J Clin Oncol* **9**: 1749–1756

Cote RJ, Beattie EJ, Chaiwun B, Shi SR, Harvey J, Chen SC, Sherrod AE, Groshen S and Taylor CR (1995) Detection of occult bone marrow micrometastases in patients with operable lung carcinoma. *Ann Surg* **4**: 415–423

Cote RJ, Houchens DP, Hitchcock CL, Saad AD, Nines RG, Greenson JK, schneebaum S, Arnold MW and Martin EW Jr (1996) Intraoperative detection of occult colon cancer micrometastases using 125 I-radiolabeled monoclonal antibody CC49. *Cancer* **77**: 613–620

Diel IJ, Kaufmann M, Costa SD, Holle R, von Minckwitz G, Solomayer EF, Kaul S and Basetert G (1996) Micrometastatic breast cancer cells in bone marrow at primary surgery: prognostic value in comparison with nodal status. *J Natl Cancer Inst* **88**: 1652–1658

Dutrillaux B, Gerbault-Seureau M and Zafrani B (1990) Characterization of chromosomal anomalies in human breast cancer. A comparison of 30 paraploid cases with few chromosome changes. *Cancer Genet Cytogenet* **49**: 203–217

Eaton MC, Hardingham JE, Kotasek D and Dobrovik A (1997) Immunobead RT-PCR: a sensitive detection method for detection of circulating tumor cells. *Biotechniques* **22**: 100–105

Engel H, Friedrich J, Kleespies C, Kurbacher CM, Schöndorf T, Grecu O, Kolhagen H and Mallmann P (1998) Detection of chromosomal aberrations in tumor cells and tumor infiltrating lymphocytes by molecular cytogenetics in patients with gynecological cancer. *Cancer Genet Cytogenet* **106**: 159–165

Fernandez JL, Goyanes V, Lopez-Fernandez C, Buno I and Gosalvez J (1996) Quantification of C-ERB-B2 gene amplification in breast cancer cells using fluorescence in situ hybridization and digital image analysis. *Cancer Genet Cytogenet* **86**: 18–21

Fiegl M, Tueni C, Schenk T, Jakesz R, Gnat M, Reiner A, Rudas M, Pirc-Danoewinata H, Marosi C and Huber H (1995) Interphase cytogenetics reveals a high incidence of aneuploidy and intra-tumour heterogeneity in breast cancer. *Br J Cancer* **72**: 51–55

Franklin WA, Shpall EJ, Archer P, Johnston CS, Garza-Williams S, Hami L, Bitter MA, Bast RC and Jones RB (1996) Immunocytochemical detection of breast cancer cells in marrow and peripheral blood of patients undergoing high dose chemotherapy with autologous stem cell support. *Breast Cancer Res Treat* **41**: 1–13

Geleick D, Müller H, Matter A, Torhorst J and Regenass U (1990) Cytogenetics of breast cancer. *Cancer Genet Cytogenet* **46**: 217–229

Harbeck N, Schwarze S, Schüren E, Yamamoto N, Moniwa N, Schmitt M, Dettmar P, Nathrath W, Jänicke F, Höfler H and Graeff H (1995) Model system for isolation of competent ovarian carcinoma cells from fresh tumor tissue by magnetic cell separation technique (MACS). *Internat J Oncol* **6**: 1249–1254

Hardingham JE, Kotasek D, Farmer B, Butler RN, Mi JX, Sage RE and Dobrovic A (1993) Immunobead-PCR: a technique for the detection of circulating tumor cells using immunomagnetic beads and the polymerase chain reaction. *Cancer Res* **53**: 3455–3458

Hardingham JE, Kotasek D, Sage RE, Eaton MC, Pascoe VH and Dobrovic A (1995) Detection of circulating tumor cells in colorectal cancer by immunobead_PCR is a sensitive prognostic marker for relapse of disease. *Mol Med* **1**: 789–794

Helfrich W, ten Poele R, Meersma GJ, Mulder NH, de Vries EJ, de Leij L and Smit EF (1997) A quantitative reverse transcriptase polymerase chain reaction-based assay to detect carcinoma cells in peripheral blood. *Br J Cancer* **76**: 29–35

Hildebrandt M, Mapara MY, Korner IJ, Bargou RC, Moldenhauer G and Dorken B (1997) Reverse transcriptase-polymerase chain reaction (RT-PCR)-controlled immunomagnetic purging of breast cancer cells using the magnetic cell separation (MACS) system: a sensitive method for monitoring purging efficiency. *Exp Hematol* **25**: 57–65

Ichikawa D, Hashimoto N, Hoshima M, Yamaguchi T, Sawai K, Nakumura Y, Takahashi T, Abe T and Inazawa J (1996) Analysis of numerical aberrations of specific chromosomes by fluorescence in situ hybridization as a diagnostic tool in breast cancer. *Cancer* **77**: 2064–2069

Ishikawa T, Kobayashi M, Mai M, Suzuki T and Ooi A (1997) Amplification of the c-erbB-2 (Her-2/neu) gene in gastric cancer cells. Detection by fluorescence in situ hybridization. *Am J Pathol* **151**: 761–768

Kallioniemi OP, Kallioniemi A, Kursiu W, Thor A, Chen LC, Smith HS, Waldman FM, Pinkel D and Gray JW (1992) ERBB2 amplification in breast cancer analyzed by fluorescence in situ hybridization. *Proc Natl Acad Sci USA* **89**: 5321–5325

Kiechle-Schwarz M, Decker HJH, Berger CS, Fiebig CS and Sandberg AA (1991) Detection of monosomy in interphase nuclei and identification of marker chromosomes using biotinylated alpha-satellite DNA probes. *Cancer Genet Cytogenet* **51**: 23–33

Kruger W, Krzizanowski C, Holweg M, Stockschlader M, Kroger N, Jung R, Mross K, Jonat W and Zander AR (1996) Reverse transcriptase/polymerase chain reaction detection of cytokeratin-19 mRNA in bone marrow and blood of breast cancer patients. *J Cancer Res Clin Oncol* **122**: 679–686

Kruger WH, Stockschlader M, Hennings S, Aschenbrenner M, Gruber M, Gutensohn K, Loliger C, Gieseking F, Jonat W and Zander AR (1996) Detection of cancer cells in peripheral blood stem cells of women with breast cancer by RT-PCR and cell culture. *Bone Marrow Transplant* **18**: 18–20

Kvalheim G (1996) Detection of occult tumour cells in bone marrow and blood in breast cancer patients – methods and clinical significance. *Acta Oncol* **35**: 13–18

Le Beau MM (1993) Detecting genetic changes in human tumor cells: Have scientists 'gone fishing?' *Blood* **81**: 1979–1983

Luppi M, Morselli M, Bandieri E, Federico M, Marasca R, Barozzi P, Ferrari MG, Savarino M, Frassoldati A and Torelli G (1996) Sensitive detection of circulating breast cancer cells by reverse-transcriptase polymerase chain reaction of maspin gene. *Ann Oncol* **7**: 619–624

Mapara MY, Körner IJ, Hildebrandt M, Bargou R, Krahl D, Reichardt P and Dörken B (1997) Monitoring of tumor cell purging after highly enriched efficient immunomagnetic selection of CD34 cells from leukapheresis products in breast cancer patients: Comparison of immunocytochemical tumor cell staining and reverse transcriptase-polymerase chain reaction. *Blood* **89**: 337–344

Martin VM, Siewert C, Scharl A, Harms T, Heinze R, Ohl S, Radbruch A, Miltenyi S and Schmitz J (1998) Immunomagnetic enrichment of disseminated epithelial tumor cells from peripheral blood by MACS. *Exp Hematol* **26**: 252–264

Micale MA, Visscher DW, Gulino SE and Wolman SR (1994) Chromosomal aneuploidy in proliferative breast disease. *Human Pathol* **25**: 29–35

Miltenyi S, Muller W, Weichel W and Radbruch A (1990) High gradient magnetic cell separation with MACS. *Cytometry* **11**: 231–238

Moscinski LC, Trudeau WL, Fields KK and Elfenbein GJ (1996) High-sensitive detection of minimal residual breast carcinoma using polymerase chain reaction and primers for cytokeratin 19. *Diagn Mol Pathol* **5**: 173–180

Muller P, Weckermann D, Riethmuller G and Schlimok G (1996) Detection of genetic alterations in micrometastatic cells in bone marrow of cancer patients by fluorescence in situ hybridization. *Cancer Genet Cytogenet* **88**: 8–16

Naume B, Borgen E, Beiske K, Herstad TK, Ravnas G, Renolen A, Trachsel S, Thrane-Steen K, Funderud S and Kvalheim G (1997) Immunomagnetic techniques for the enrichment and detection of isolated breast carcinoma cells in bone marrow and peripheral blood. *J Hematother* **6**: 103–114

Noguchi S, Aihara T, Motomura K, Inaji H, Imaoka S and Koyama H (1996) Detection of breast cancer micrometastases in axillary lymph nodes by means of reverse transcriptase-polymerase chain reaction. Comparison between MUC1 mRNA and keratin 19 mRNA amplification. *Am J Pathol* **148**: 649–656

Pantel K (1996) Detection of minimal residual disease in patients with solid tumors. *J Hematother* **5**: 359–367

Persons DL, Hartmann LC, Herath JF, Keeney GL and Jenkins RB (1994) Fluorescence in situ hybridization analysis of trisomy 12 in ovarian tumors. *Am J Clin Pathol* **102**: 775–779

Press MF, Bernstein L, Thomas PA, Meisner LF, Zhou JY, Ma Y, Hung G, Robinson RA, Harris C, El-Naggar AK, Slamon DJ, Phillips RN, Ross JS, Wolman SR and Flom KJ (1997) HER-2/neu gene amplification characterized by fluorescence in situ hybridization: poor prognosis in node-negative breast carcinomas. *J Clin Oncol* **15**: 2894–2904

*© 1999 Cancer Research Campaign*

Revillion F, Bonneterre J and Peyrat JP (1998) ERBB2 oncogene in human breast cancer and its clinical significance. *Eur J Cancer* **34**: 791–808

Ross AA (1998) Minimal residual disease in solid tumor malignancies: a review. *J Hematother* **7**: 9–18

Sauter G, Feichter G, Torhorst J, Moch H, Novotna H, Wagner U, Durmuller U and Waldman FM (1996) Fluorescence in situ hybridization for detecting erbB-2 amplification in breast tumor fine needle aspiration biopsies. *Acta Cytol* **40**: 164–173

Schildkraut JM, Collins NK, Dent GA, Tucker JA, Barret JC, Berchuck A and Boyd J (1995) Loss of heterozygosity on chromosome 17q11–21 in cancers of women who have both breast and ovarian cancer. *Am J Obstet Gynecol* **172**: 908–913

Schoenefeld A, Luqmani Y, Smith D, O'Reilly S, Shousha S, Sinnet HD and Coombes RC (1994) Detection of breast cancer micrometatases in axillary lymph nodes by using polymerase chain reaction. *Cancer Res* **54**: 2986–2990

Schoenfeld A, Kruger KH, Gomm J, Sinnett HD, Gazet JC, Sacks N, Bender HG, Luqmani Y and Coombes RC (1997) The detection of micrometastases in the peripheral blood and bone marrow of patients with breast cancer using immunohistochemistry and reverse transcriptase polymerase chain reaction for keratin 19. *Eur J Cancer* **33**: 854–861

Smith B, Selby P, Southgate J, Pittman K, Bradley C and Blair GE (1991) Detection of melanoma cells in peripheral blood by means of reverse transcriptase and polymerase chain reaction. *Lancet* **338**: 1227–1229

Traystman MD, Cochran GT, Hake SJ, Kuszynski CA, Mann SL, Murphy BJ, Pirruccello SJ, Zuvanich E and Sharp JG (1997) Comparison of molecular cytokeratin 19 reverse transcriptase polymerase chain reaction (CK19 RT-PCR) and immunocytochemical detection of micrometastatic breast cancer cells in hematopoietic harvests. *J Hematother* **6**: 551–561

Visscher DW, Wallis T and Ritchie CA (1995) Detection of chromosome aneuploidy in breast lesions with fluorescence in situ hybridization: comparison of whole nuclei to thin tissue sections and correlation with flow cytometric DNA analysis. *Cytometry* **21**: 95–100

Visscher DW, Wallis TL and Crissmann JD (1996) Evaluation of chromosome aneuploidy in tissue sections of preinvasive breast carcinomas using interphase cytogenetics. *Cancer* **77**: 315–320

Vrendenburgh JJ, Silva O, Tyer C, DeSombre K, Abou-Ghalia A, Cook M, Layfied L, Peters WP and Bast RC Jr (1996) A comparison of immunohistochemistry, two-color immunofluorescence, and flow cytometry with cell sorting for the detection of micrometastatic breast cancer in the bone marrow. *J Hematother* **5**: 57–62

Xu J and Wang N (1994) Identification of chromosomal structural alterations in human ovarian carcinoma cells using combined GTG-banding and repetitive fluorescence in situ hybridization (FISH). *Cancer Genet Cytogenet* **74**: 1–7

*© 1999 Cancer Research Campaign*
